# Development and validation of a predictive score for chemoresistance in high-grade osteosarcoma at baseline

**DOI:** 10.3389/fmed.2025.1588302

**Published:** 2025-07-04

**Authors:** Thanat Kanthawang, Nuttaya Pattamapaspong, Jongkolnee Settakorn, Pattira Boonsri, Pimpisa Teeyakasem, Phichayut Phinyo, Dumnoensun Pruksakorn

**Affiliations:** ^1^Department of Radiology, Faculty of Medicine, Chiang Mai University, Chiang Mai, Thailand; ^2^Department of Pathology, Faculty of Medicine, Chiang Mai University, Chiang Mai, Thailand; ^3^Department of Radiology, Faculty of Medicine, Prince of Songkla University, Songkhla, Thailand; ^4^Department of Orthopedic, Faculty of Medicine, Chiang Mai University, Chiang Mai, Thailand; ^5^Center of Multidisciplinary Technology for Advanced Medicine (CMUTEAM), Faculty of Medicine, Chiang Mai University, Chiang Mai, Thailand; ^6^Department of Biomedical Informatics and Clinical Epidemiology (BioCE), Faculty of Medicine, Chiang Mai University, Chiang Mai, Thailand; ^7^Center for Clinical Epidemiology and Clinical Statistics, Faculty of Medicine, Chiang Mai University, Chiang Mai, Thailand

**Keywords:** clinical prediction rule, osteosarcoma, chemotherapy, magnetic resonance imaging, mortality, chemoresistance, tumor necrosis

## Abstract

**Objective:**

Histological tumor necrosis is the current indicator for the response of osteosarcoma after neoadjuvant chemotherapy. Chemoresistant tumors require close monitoring and adjustment of treatment. We aimed to develop a prediction score for chemoresistance in newly diagnosed osteosarcoma patients underwent neoadjuvant chemotherapy.

**Materials and methods:**

Data from a registry-based cohort of high-grade osteosarcoma patients treated with neoadjuvant chemotherapy between January 2008 and October 2023 were used. Histological necrosis from surgical specimens was the reference standard. Clinical and MRI parameters at baseline were derived by risk regression analysis.

**Results:**

From 139 patients, 93 (66.91%) were classified as chemoresistant (histological necrosis <90%). The model included four predictors: age >40 years, initial metastasis, tumor volume (≤150 ml, > 150–400, or > 400 ml), and pre-chemotherapy tumor necrosis >50%. The AuROC of the model was 0.76 (95% CI 0.68–0.85) and well-calibrated. Internal validation using a bootstrap technique showed consistent AuROC results. The prediction score ranged from 0 to 8, with a score of 0–2 indicating low probability (positive LHR = 0.45) and a score of 3–8 indicating high probability (positive LHR = 2.56) of chemoresistance.

**Conclusion:**

High-grade osteosarcoma patients with a prediction score of 3–8 have a high probability of chemoresistance. This score could be used for risk communication and tailoring management at diagnosis.

## 1 Introduction

Osteosarcoma (OS) is the predominant form of primary bone cancer, and chemotherapy has significantly improved survival rates (1). The standard approach involves neoadjuvant chemotherapy followed by surgical tumor removal, leading to increased long-term survival (2–4). Despite improvement of treatment, chemotherapy responses vary with nearly half of OS patients showing unfavorable outcomes (5, 6). Intensified chemotherapy can cause adverse effects, and suboptimal responses pose challenges in surgical planning (7, 8). Therefore, predicting responsiveness before surgery is crucial for personalized treatment plans.

Currently, the gold standard for evaluating the response to pre-operative chemotherapy in OS is histological assessment of tumor necrosis on resected specimens (9). Recognized as the most reliable prognostic factor in OS, this method significantly influences management decisions (5, 10). Poor responder is a strong predictor of local recurrence, metastases and overall survival (11). In detail, high disease-free survival rates are observed in chemoresponders, making them suitable candidates for limb salvage surgery. In contrast, poor responders require aggressive radical surgery and a modification of the postoperative chemotherapy regimen (4). However, the practical application of histological response is limited because surgical specimens are only accessible after completing the entire course of pre-operative neoadjuvant chemotherapy.

Several studies have explored the correlation between clinical parameters, laboratory examinations, and histologic response in OS, results remain inconclusive (2, 5, 12, 13). Conventional magnetic resonance imaging (MRI) remains the predominant imaging modality in routine clinical practice, crucial for assessing tumor extent and facilitating surgical planning (14, 15). Although conventional MRI alone can predict chemoresistant OS, its performance is fair to moderate (16). Recognizing that treatment outcomes are multifactorial and influenced by diverse factors, there is a necessity for an evaluation method that combines clinical and imaging features to predict the chemotherapeutic effect for OS patients.

Identifying patients at risk of a poor response to chemotherapy in the early stages of the disease can assist in selecting patients for tailored management, including closed monitoring or early imaging reassessment before completing neoadjuvant chemotherapy, and mitigate complications linked to ineffective chemotherapy (17, 18). A few studies have utilized artificial intelligence based on baseline MRI or positron emission tomography (PET) to develop models or clinical prediction rules for predicting histologic chemoresistance in OS (19–25). Others have incorporated early changes observed in follow-up MRIs along with clinical parameters or additional imaging modalities, such as angiograms, to predict chemoresistance in OS (13, 26, 27). However, these approaches have limited use in routine clinical practice due to the need for other specialized software or additional imaging modalities.

Some imaging features or clinical manifestations, when coexisted, may help predict tumor aggressiveness and the risk of poor response to chemotherapy. Although potential to be widely used in routine clinical practice, the prediction score using combined clinical and conventional MRI features has not been comprehensively examined. Consequently, this study aimed to develop a clinical prediction score integrating clinical and baseline conventional MRI parameters to predict chemoresistance in high-grade OS patients underwent neoadjuvant chemotherapy, using histological response as the reference standard.

## 2 Materials and methods

### 2.1 Study design and patient cohort

This retrospective study involved the development and internal validation of multivariable clinical prediction rules based on a registry-based cohort using baseline clinical and MRI parameters. All OS cases in the cancer registry of our institution from January 2008 to October 2023 were assessed for eligibility. The study protocol was in accordance with the Declaration of Helsinki and was approved by the institutional review boards of our institution (RAD-2565-08966). As the data were retrospectively collected, informed consent was waived. All patient data used were anonymized and kept confidential only accessible to the research team.

All patients included in this study were those diagnosed with high-grade OS (Enneking staging IIB-III), who underwent neoadjuvant chemotherapy followed by surgery at our university tertiary care centers, yielding 191 patients. Patients without baseline MRI at the time of diagnosis (27 patients) were excluded. Other exclusion criteria of the 25 cases were post-treated tumors by radiation therapy or surgery (11 patients), secondary OS (10 patients), and poor image quality (4 patients). Finally, 139 patients were included in this study.

**Figure 1:**
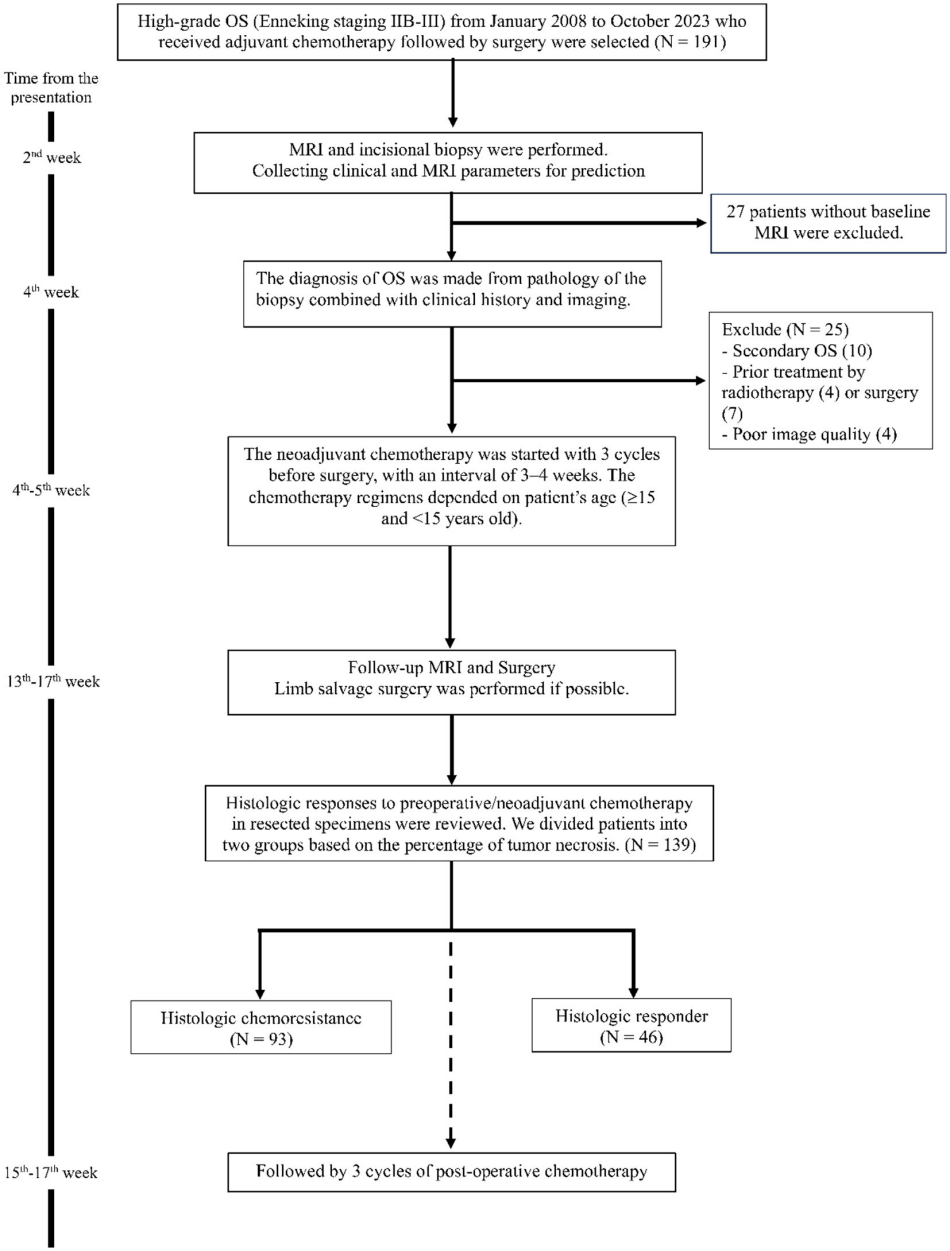
Patient flow diagram.

Patients diagnosed OS have consistently followed a standardized protocol at our institution since 1996 with change of chemotherapy regimens for pediatric patient in 2014. Within 2 weeks of the initial presentation, an MRI was conducted, and an incisional biopsy was performed within the subsequent week. The pathological diagnosis of OS was confirmed within 2 weeks following the biopsy. The multidisciplinary team promptly initiated the treatment plan within 1 week of diagnosis, involving the commencement of three cycles of neoadjuvant chemotherapy followed by surgery. Whenever feasible, limb salvage surgery was prioritized. Notably, two neoadjuvant chemotherapy regimens were employed, stratified by patient age (< 15 vs. ≥15 years) (28). For adolescent and adult patients (≥15 years of age), the first-line chemotherapy consisted of Doxorubicin (50 mg/m^2^) and Cisplatin (80 mg/m^2^) administered at an interval of 3–4 weeks for 3 cycles as neoadjuvant therapy, followed by an additional three cycles for adjuvant therapy. For pediatric patients (< 15 years old), the first-line chemotherapy included Carboplatin (400 mg/m^2^/dose on day 1) and Doxorubicin (20 mg/m^2^/day on day 1–3), also administered at an interval of 3–4 weeks for three cycles as neoadjuvant therapy, followed by 3–4 cycles of Carboplatin and Doxorubicin for adjuvant therapy before 2014. Since 2014, high-dose Methotrexate (12 gm/m^2^/day) has been added to the treatment protocol for pediatric patient (29). Figure 1 provides the timeline illustrating the patient management and selection process.

### 2.2 Definition of histologic response

Following the surgical resection of the tumor, specimens were forwarded to the pathology laboratory for histological evaluation. Each specimen underwent serial cutting, with the largest cut surface selected and fixed in 10% buffered formalin. The entire cut surface was sectioned into small pieces and embedded in paraffin blocks. For specimens with bony hard consistency, decalcification preceded the embedding process. Paraffin blocks were then sectioned into 3–4 microns thick slices and stained with hematoxylin and eosin. The assessment of tumor necrosis percentage on each slide was used to calculate the average tumor necrosis. Patients were stratified into two groups based on the percentage of tumor necrosis: histologic responders (≥90% necrosis) and histologic chemoresistance (< 90% necrosis) (30). Histologic responses to preoperative chemotherapy in resected specimens were meticulously reviewed by a single musculoskeletal pathologist.

### 2.3 Data collection and predictors

We collected baseline clinical data from our tumor registry database, including age, gender, anthropometric measurements (weight, height, body mass index and body surface area), Enneking staging (31), pathologic subtype, location, pathologic fracture, initial metastasis, serum alkaline phosphatase (ALP), and types of surgery. Tumor location was categorized into axial, upper extremity (including scapula), and lower extremity. Because growth influences ALP expression, 150 U/L was considered as the upper normal serum ALP limit in patients < 18 years old, and 110 U/L in those 18 years or older (32). The initial metastasis status detected by imaging investigation (CT chest, bone scan and screening whole limb MRI) can be either pulmonary, bone metastasis or both. For pulmonary metastasis, patients were categorized as having pulmonary metastasis from baseline CT scans if they had a single nodule measuring more than 10 millimeters (mm) in diameter, or three or more (multiple) nodules measuring five to nine mm in diameter, or a calcified pulmonary nodule in pediatric patients. These criteria for pulmonary metastasis are supported by previous literatures (33, 34).

All MRIs were newly reviewed on Picture Archiving and Communication System (PACS) workstations by a board-certified musculoskeletal radiologist with 9 years of experience (T.K.), who was blinded to clinical information and histologic response. The MRI parameters were selected based on significant parameters from previous study (16), including a tumor volume exceeding 150 milliliters (ml), the presence of tumor necrosis covering more than 50% of the tumor area, and intra-articular extension. For tumor sizing, we chose to use tumor volume instead of the maximal axial diameter, as indicated in previous studies, which demonstrated that tumor volume is the only independent predictor (16, 35). In this study, we did not include the extension of peritumoral soft tissue edema, even though it showed significant results in previous studies (16–18). This decision was made because several tumor related complications, such as deep vein thrombosis or pathologic fracture, can contribute to soft tissue edema in MRI. Additionally, previous studies have indicated that peritumoral soft tissue edema in bone tumors less likely represents tumor cells (36, 37). We also collected information on presence of pathological fractures by correlating data from MRI with plain radiographs.

Tumor volume was calculated using the elliptical formula (0.53 × width × cross-sectional length × height). Measurements of tumor volume and grading of tumor necrosis were performed on post-contrast studies correlated with unenhanced T1W and T2W sequences, such as using T1W images for intramedullary extent. The extension of the tumor into the synovial space or along the cruciate ligaments was defined as signs of intra-articular extension. The inter-observer reproducibility for each MRI parameter was evaluated based on a previous study, showing an ICC range of 0.69–0.98, indicating moderate to excellent agreement between reviewers (16).

All patients underwent MRI at 1.5 or 3 Tesla, using machines from different vendors depending on availability in the referral hospitals. The majority (95 out of 139; 68.35%) of MRI studies were conducted at our institution using either MRI Signa 1.5T Excite HD or MRI Signa 1.5T HDxt or MRI Signa Pioneer 3T (GE Healthcare, Best, Netherlands). While 44 studies (31.65%) were performed at other centers before referral to the musculoskeletal sarcoma multidisciplinary team (MDT) meeting. Among these, 33 MRI studies were conducted at 1.5T (Magnetom, Siemens Healthcare, Erlangen, Germany). Technical parameters are detailed in Supplementary Tables S1, S2. However, technical parameters were unavailable for 11 patients. Sequences used included at least T1-weighted (T1W) and fluid-sensitive sequences (STIR or fat-suppressed T2W images) in the axial plane, along with at least one orthogonal view (sagittal and coronal views). T1W and STIR sequences of the affected extremity were also obtained for screening of skip metastases. Contrast-enhanced, fat-suppressed T1W images were performed in sagittal, coronal, and axial planes.

Finally, a total of nine factors were included in univariable regression analysis, comprising age >40 years, female gender, axial location, initial metastasis, elevated ALP, tumor volume, presence of tumor necrosis, intra-articular extension, and pathologic fracture. The selection of predictors was based on the availability of predictors at the time of prediction, clinical expertise, and previous literature. Each predictor was categorized at a generally accepted cutoff point, according to those in the literature.

### 2.4 Study size estimation

We employed two statistical approaches in estimating the study size. First, we estimated the study size based on the comparison of the mean or proportion of potentially significant predictors (i.e., tumor volume >150 ml) between groups of patients with and without a histologic response using the previously reported data (16). The proportion of OS patients with a histologic response was 0.5, whereas those with histologic chemoresistance was 0.75 (16). Given a ratio between histologic response and non-response of 0.5/0.75, a total study size of 136 patients was required to achieve an 80% statistical power and a two-sided alpha error of 0.05. Second, we followed the general suggestion of rule of thumb that at least 10 events are needed for one predictor variable in the logistic model (38). As we anticipated that our model would include up to 9 predictor variables, a total of 90 chemoresistant events were required. Thus, based on the proportion of histologic non-response in OS patients at 0.75 (16), we planned to include at least 120 patients.

### 2.5 Statistical analysis and score development

All statistical analyses were performed using Stata 17 (StataCorp, College Station, Texas, USA). A *P*-value < 0.05 was considered a statistically significant difference. Categorical variables were presented as frequencies and percentages and continuous variables as mean and standard deviation for normal distribution data, median and inter-quartile ratio for non-normal distribution data. The differences between histologic responder and histologic chemoresistance groups were assessed using Mann–Whitney *U*-test or *T*-test (continuous variables) and Fisher's exact test (categorical variables). To examine the discriminative ability of potential predictors, we estimated the area under the receiver operating characteristic (AuROC) curve for each predictor.

The selection of potential predictors for the prognostic score was determined through the statistical significance of the univariable analysis. All predictors with a univariable *P*-value < 0.20 were incorporated into a multivariable logistic model. Subsequently, predictors with a *P*-value exceeding 0.05 and an odds ratio close to 1.0 were sequentially eliminated from the model in a backward fashion. Each predictor in the final model received a weighted score calculated by dividing the logit coefficient of each predictor by the lowest coefficient in the model. Consequently, the predictor with the lowest logit coefficient value was assigned a score of one. The results of these divisions were rounded up to whole numbers. The sum of the total score for each patient in the dataset was then utilized to assess predictive performance. Evaluation of the score's performance included considerations of model discrimination and calibration. Discriminative ability was determined based on the AuROC. Calibration was assessed through the score calibration plot and the Hosmer-Lemeshow goodness-of-fit test, where the plot contrasted the sum of the total score with the observed proportion of histologic chemoresistance within each score stratum.

The patients in the dataset were categorized into two risk groups based on their scores: low risk and high risk. This decision was made to help clinicians prioritize patients for early and more aggressive intervention. The determination of score cutoff points was based on achieving an optimal balance between sensitivity and specificity (Supplementary Table S3). Positive likelihood ratios (LHR+) were calculated, along with their corresponding 95% confidence intervals (CI), to indicate the predictive ability of each score category for histologic chemoresistance. An LHR+ < 1 signified significantly lower odds of chemoresistance compared to the general population, while an LHR+ higher than 1 indicated significantly higher odds of chemoresistance. The discriminative ability of the score categories was reassessed by examining whether the confidence intervals of LHR+ for each category overlapped. To gauge the optimism of the derived score in predicting chemoresistance, an internal validation was conducted using bootstrap resampling procedures with 500 replicates.

## 3 Results

From the 139 patients, there were 93 patients with histologic chemoresistance with an estimated incidence of 66.91%. The median age in all patients was 16 years (range from 2 to 75) with 69 patients (49.64%) receiving Carboplatin and Doxorubicin chemotherapy owing to an age < 15 years. Most of the patients had histologically conventional OS (112/139; 80.58%) and 44 patients (31.65%) had regional or distant metastasis (Enneking staging III).

In detail, 44 out of 46 (95.65%) patients with histologic responder group and 68 out of 93 (73.12%) patients with histologic chemoresistance group had conventional OS (*P*-value = 0.006). Of the 93 patients with chemoresistance, 15 (16.13%) had chondroblastic OS and 8 (8.60%) had telangiectasis OS, whereas only 1 patient in the responder group had chondroblastic OS (*P* = 0.006). Additionally, 6 out of 46 (13.04%) vs. 21 out of 93 (22.58%) patients had metastasis in each group, respectively (*P* = 0.009). Detailed analysis of metastasis patterns revealed that 30 patients (3 responders, 27 chemoresistant) had lung-only metastases, 14 (4 responders, 10 chemoresistant) had bone-only metastases, and 14 (2 responders, 12 chemoresistant) had both lung and bone metastases.

There were no statistically significant differences in gender, weight, height, body surface area and BMI among these two groups. Limb-salvage surgery was performed in 95 patients −43 (93.48%) histologic responders and 52 (55.91%) patients with chemoresistance. The remaining patients underwent amputation.

Several baseline clinical and MRI factors were identified as significant predictors in univariable analysis (Table 1) and were subsequently included in the multivariable model (Table 2). After backward elimination of non-significant predictors from the multivariable logistic regression model, the following factors were identified as significant predictors for histologic chemoresistance in high-grade OS patients: age ( ≤ 40 and >40 years), initial metastasis, tumor volume (≤ 150, 150–400, and >400 ml), and the presence of tumor necrosis >50% (Table 2). After score transformation, the sum of the derived score ranges from 0 to 8, with an overall mean score of all patients at 2.91 ± 2.19. The average score differed significantly between histologic responder and histologic chemoresistance groups (1.65 ± 1.89 vs. 3.54 ± 2.07, *P* < 0.001). A cutoff point of 3 was identified to classify all included patients into low and high risk, achieving a sensitivity of 66.67% and a specificity of 73.91%. Supplementary Table S3 shows the sensitivity and specificity of each score cutoff point.

**Table 1:** Clinical and MRI predictor variables of the cohort.

**Characteristics**	**Response to chemotherapy (*****N*** = **139)**		**OR (95%CI)**	*P*-value^*^	**AuROC (95%CI)**
	**Chemoresistance (*****N*** = **93)**	**Responder (*****N*** = **46)**			
Age					
≤ 40 years	75 (80.65%)	44 (95.65%)	Reference		
>40 years	18 (19.35%)	2 (4.35%)	5.28 (1.17–23.84)	0.031	0.58 (0.52–0.63)
Female gender	44 (47.31%)	15 (32.61%)	1.86 (0.89–3.88)	0.101	0.57 (0.49–0.66)
Axial location	14 (15.05%)	2 (4.35%)	3.90 (0.85–17.95)	0.081	0.55 (0.51–0.60)
Initial metastasis	37 (39.78%)	7 (15.22%)	3.68 (1.49–9.10)	0.005	0.62 (0.55–0.70)
Elevated ALP	68 (74.73%)	33 (71.74%)	1.16 (0.52–2.58)	0.708	0.51 (0.43–0.59)
Tumor volume (ml)					
≤ 150 ml	19 (20.43%)	19 (41.30%)	Reference		0.66 (0.57–0.75)
>150–400 ml	33 (35.48%)	18 (39.13%)	1.83 (0.78–4.32)	0.166	
>400 ml	41 (44.09%)	9 (19.57%)	4.56 (1.74–11.92)	0.002	
Presence of tumor necrosis >50%	52 (55.91%)	11 (23.91%)	4.04 (1.83–8.91)	0.001	0.66 (0.58–0.74)
Intra-articular extension	53 (56.99%)	19 (41.30%)	1.88 (0.92–3.85)	0.083	0.58 (0.49–0.67)
Pathologic fracture	12 (12.90%)	3 (6.52%)	1.94 (0.51–7.35)	0.329	0.53 (0.47–0.59)

* Logistic regression analysis.

Numbers are *n* (%).

AuROC, area under the receiver operating characteristic curve; OR, Odds ratio; CI, confidence interval; ALP, alkaline phosphatase; ml, millimeters.

**Table 2:** Risk score derivation using multivariable logistic regression coefficients.

**Predictors**	**OR**	**95%CI**	***P*-value**	**β-coefficients**	**Item score**
Age					
≤ 40 years	1	Reference	-	-	0
>40 years	2.89	0.58–14.30	0.194	1.06	2
Initial metastasis	2.75	1.10–7.18	0.038	1.01	2
Tumor volume (ml)					
≤ 150 ml	1	Reference	-	-	0
>150–400 ml	1.58	0.63–3.94	0.326	0.45	1
>400 ml	2.76	0.98–7.78	0.054	1.02	2
Tumor necrosis >50%	2.75	1.18–6.42	0.019	1.01	2

OR, Odds ratio; CI, confidence interval; β, beta-coefficient.

We present the incidence of histologic chemoresistance and the LHR+ for each category in Table 3. The predictive performance of the scoring system was evaluated by stratifying patients into low- and high-risk categories based on their total score (range: 0–8). Of the 139 patients, 65 (46.76%) were classified as low-risk (score 0–2), and 74 (53.24%) as high-risk (score 3–8). Among the low-risk group, 31 patients (47.69%) had histologic chemoresistance and 34 (52.31%) were responders, resulting in an LHR^+^ of 0.45 (95% CI: 0.24–0.86). In contrast, the high-risk group showed a significantly different distribution, with 62 patients (83.78%) exhibiting chemoresistance and only 12 (16.22%) being responders. The corresponding LHR^+^ was 2.56 (1.21–5.71).

**Table 3:** Distribution of histologic chemoresistance vs. responders and likelihood ratio of chemoresistance (LHR+) in low- and high-risk categories.

**Risk categories**	**Score**	Total^*^(*N* = 139)	Chemoresistance^**^(*N* = 93)	Responder^**^(*N* = 46)	**LHR+**	**95%CI**
Low	0–2	65 (46.76%)	31 (47.69%)	34 (52.31%)	0.45	0.24–0.86
High	3–8	74 (53.24%)	62 (83.78%)	12 (16.22%)	2.56	1.21–5.71
Mean ± SD			3.54 ± 2.07	1.65 ± 1.89		

* Column percentage.

** Row percentage.

Numbers are n (%) or mean ± SD.

CI, confidence interval; LHR+, positive likelihood ratio; SD, standard deviation.

**Figure 2:**
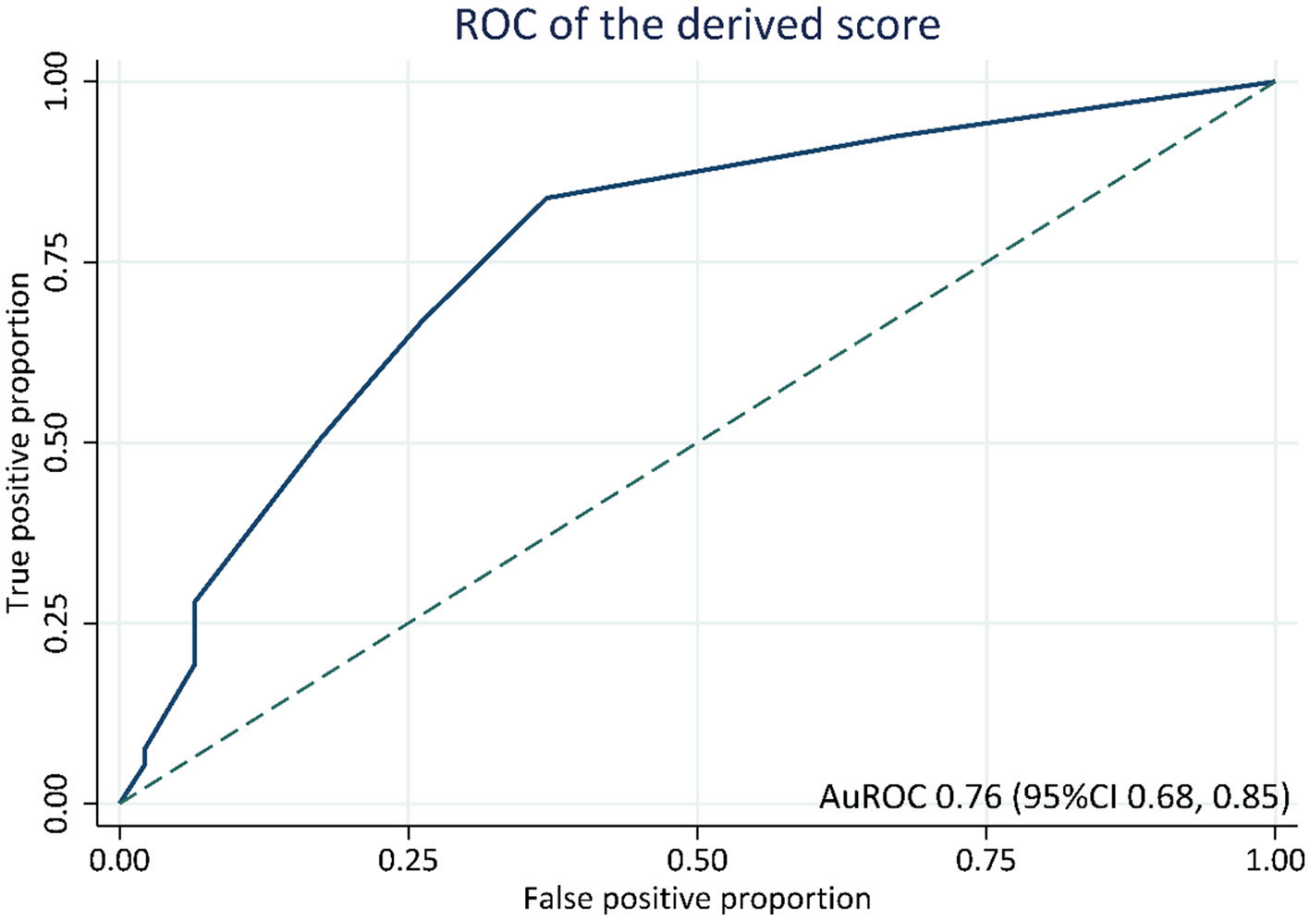
ROC curve for prediction of histologic chemoresistance by the derived score. AuROC, area under the receiver operating characteristic curve; CI, confidence interval.

**Figure 3:**
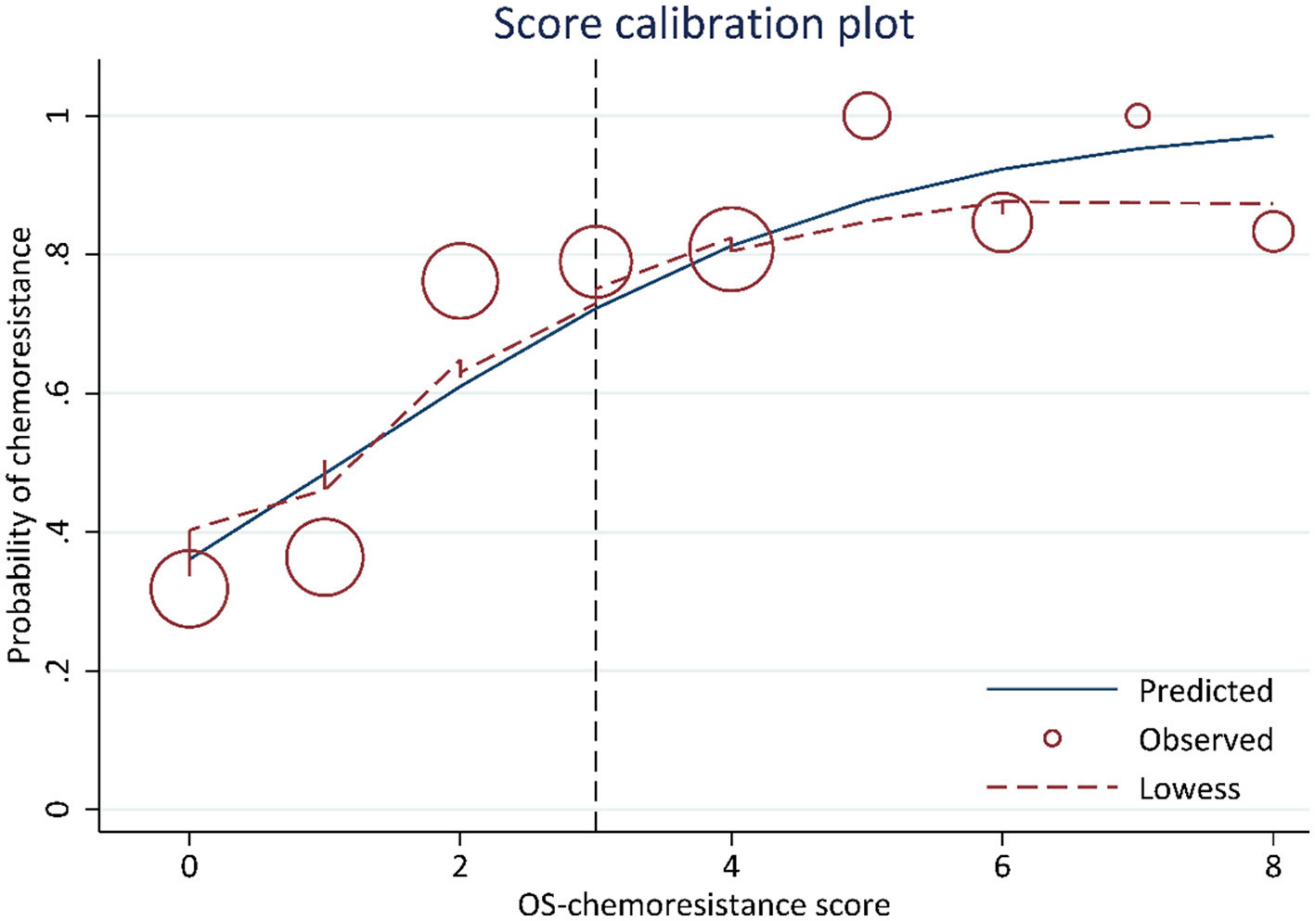
Predicted risk histologic chemoresistance by score (solid line) and observed risk histologic chemoresistance (hollow circles).

The score demonstrated an acceptable ability to discriminate between histologic responder and chemoresistance groups based on an apparent AuROC at 0.76 (95% CI 0.68–0.85; Figure 2). After internal validation with the bootstrap procedure, the test AuROC remained 0.76 (95% CI 0.68–0.85). The estimated optimism of C-statistics for chemoresistance was 0.024. In terms of score calibration, the Hosmer-Lemeshow goodness-of-fit statistics revealed a non-significant result (no statistical evidence of lack-of-fit; *P* = 0.227), indicating an almost perfect fit of the model to the observed data. The score calibration plot also showed good agreement between the risk score and the observed risk of chemoresistance (Figure 3).

We conducted a *post-hoc* subgroup analysis to evaluate the performance of the scoring model across different subgroup levels, as shown in Supplementary Table S4. The model demonstrated consistent discriminative ability across tumor location, sex, and age groups, with no statistically significant differences in AuROC observed.

## 4 Discussion

In this study, we have developed and internally validated clinical decision rules for prediction of the histologic chemoresistance in patients with high-grade OS using baseline clinical and MRI factors. Four clinical and MRI predictors were included in the model, which were age >40 years, presence of initial metastasis, tumor volume and presence of tumor necrosis >50%. The scoring system delivered acceptable discrimination and good calibration. Patients with scores above 3 are considered high-risk and have a 2.56-fold increased likelihood of histologic chemoresistance. These findings may assist clinicians in stratifying patients more effectively and tailoring treatment strategies accordingly.

Neoadjuvant chemotherapy is a crucial role in OS treatment by reducing tumor bulk, facilitating surgical procedures, improving the chance of limb salvage, enabling tumor-free margins and eliminating micrometastases. Despite these advancements, OS remains a disease with poor prognosis due to its genomic complexity and significant tumor response heterogeneity to chemotherapy. Clinical trials for newly diagnosed OS patients continue to explore the feasibility of response-adapted therapy (39). Therefore, accurate pre-treatment prediction of tumor response to chemotherapy is essential for early identification of OS cases with chemoresistance. This can aid in modifying individualized treatment care regimens, such as close monitoring, early imaging reassessment, potential additional second-line chemotherapy, or early surgical intervention (17, 40).

The incidence of histologic responders in our study is 33%, which is slightly lower compared to the 45%−55.6% reported in previous literature (5, 6). The difference in chemotherapeutic protocols undoubtedly explains the heterogeneity in chemoresponsiveness across studies. Our institution's first-line regimen for both childhood and adult OS consists of adriamycin and platinum-based chemotherapy with additional high-dose methotrexate since 2014. Survival rates have been shown to be higher in series using three or more drugs in combination compared to those using two drugs (2, 5, 41, 42). Furthermore, the slightly higher incidence (31.65%) of systemic metastases in our study compared to the prior studies (~10%−20%) may account for this poorer chemotherapy response (5, 41, 42). This also supports a previous report from our group, which indicated lower survival rates compared to those from studies conducted in Western and other Asian populations (41).

Currently, there are no validated molecular biomarkers at the time of diagnosis that can predict responsiveness to chemotherapy in OS (43). Various MRI parameters, whether considered individually or integrated into a clinical prediction score, have demonstrated effectiveness in evaluating response. However, these assessments were predominantly established following a complete series of pre-operative chemotherapy. To the best of our knowledge, this is the first study to combine clinical factors and conventional MRI parameters at baseline to predict chemoresistant OS, without relying on advanced MRI (such as diffusion weighted image) or artificial intelligence techniques, making it a more practical approach. The included clinical and MRI factors investigated in our study drew upon findings from prior studies (2, 5, 12, 13, 16–18, 35).

OS demonstrates a bimodal age distribution, with 18%−30% of cases occurring in patients older than 40 years. Approximately 14% of our patients fall within this age group. Patients over 40 years old at presentation typically exhibit a poorer prognosis than younger patients due to a higher incidence of axial bone involvement and metastasis, a lower rate of chemotherapy tolerance, and resistance to chemotherapy (11, 44).

The presence of initial metastatic disease indicates a poor prognosis, with only ~20% survival rate (5, 45). However, the reasons for the poor response to chemotherapy in OS with initial metastasis are not well understood. The heightened aggressiveness of the tumor, along with the complex molecular alterations in patients with metastasis, may account for this outcome. Moreover, almost OS patients are presumed to have subclinical micrometastasis lesions at diagnosis, whereas only 15%−20% of newly diagnosed area successfully detected with metastasis (5, 11, 45). The most common site of distant spread is the lung (60%−70%), followed by bone (either skip or distant) in 20%−30% of cases (11, 45). The incidence of metastasis in our study aligned with previous literatures.

Tumor size is not only a reflection of the intrinsic biological nature of the tumor but also contributes to metastatic disease (46). The size of the tumor is associated with high cellularity and high chemoresistant clones (47). In clinical practice, delays in diagnosis or treatment that result in larger tumor volumes and initial metastasis can contribute to chemoresistance (41). At the baseline MRI, a large tumor size has been identified as a predictor of poor chemotherapy response, but the optimal method and cutoff for measurement remain subjects of debate. The ellipsoidal shape method for measuring tumor volume is widely accepted (16, 35). This measurement method represents tumor size in both axial and vertical extensions, as recommended by the ESMO-PaedCan-EURACAN Clinical Practice Guidelines (48).

Post-treatment tumor necrosis in OS has been extensively studied as an indicator of chemotherapy response, but the association between baseline necrosis on MRI and treatment response is less explored. This gap in knowledge is largely due to neoadjuvant chemotherapy being the standard of care, which limits the assessment of pre-treatment necrosis against post-treatment histopathology (46). Necrosis develops in response to treatment due to cellular death, vascular changes, and reduced perfusion (49). Larger tumors often exhibit more extensive necrosis, suggesting a relationship between tumor size and perfusion (16). Baseline MRI might underestimate necrosis due to gadolinium enhancement in granulation and fibrous tissue, neovascularization, and reactive hyperemia (22). However, this effect is likely minimal when categorizing necrotic areas into those less than or exceeding 50%, as we did in our study.

Different subtypes of OS exhibit distinct overall survival rates and responses to chemotherapy. Chondroblastic OS tends to manifest in older patients, is more commonly found in axial locations, and displays greater resistance to chemotherapy than conventional OS, as evidenced by our study, which observed a higher incidence of chondroblastic OS in the chemoresistance group (50). However, we did not include histologic subtype data from open surgical or needle biopsies in this analysis, although previous studies have reported significant findings from such data. This is because the evaluation of sclerotic tumors and the partial assessment of the entire mass from biopsy are less effective (51). Like tumor necrosis, chondroblastic components have lower perfusion, which contributes to chemoresistance, and can present as a central non-enhancing portion on imaging, making it difficult to distinguish chondroblastic subtypes from other components with sufficient reliability (47).

Our study has both strengths and limitations. In terms of strengths, firstly, the study had a large sample size, particularly given the low incidence of the tumor. Secondly, we used data from a registry-based cohort, utilizing a standardized record form, with only two cases of missing data for serum ALP. Thirdly, this score was derived using the histology from surgical specimens which is the gold standard for evaluating chemoresponsiveness and is an important predictor in the management of OS patients (5, 10). Finally, we followed a methodological and statistical standard in the derivation of the prognostic score. All factors included in the model were pre-selected based on previous clinical evidence, clinical experience, and statistical significance. The predictors included in our model are readily applicable in practice and are supported by solid theoretical foundations.

However, our study also has a few limitations. First, the MRI data were acquired from multiple centers using different scanners and technical parameters. This inter-scanner variability may impact the robustness of the imaging predictors. We addressed this by focusing on parameters less susceptible to technical variation. Future studies should further examine the impact of scanner-specific factors. Second, certain potential predictors for chemoresistance in OS, such as delayed or refused treatment, were not examined due to challenges in evaluating patient-reported onset and the reliability of this information. Finally, our study was conducted at a single tertiary care center with a specific patient population, which limits the generalizability of the prediction model. Although the model demonstrated robust internal validation, external validation remains essential. Thus, before implementation in clinical practice, further validation through prospective external studies in diverse settings is necessary.

## 5 Conclusion

Four routinely available predictors in the chemoresistance OS score showed acceptable discriminative ability and calibration in predicting chemoresistance in high-grade OS patients treated with neoadjuvant chemotherapy. The score might be applied to help clinicians stratify patients into risk groups for appropriate management and provide effective risk communication to patients and their families. We therefore emphasize the need for future prospective studies in multi-center cohorts to validate and enhance the generalizability of our prediction model across diverse clinical settings and imaging protocols.

## Data Availability

The original contributions presented in the study are included in the article/Supplementary material, further inquiries can be directed to the corresponding authors.
