# Supplementary material for: Development and validation of a predictive score for chemoresistance in high-grade osteosarcoma at baseline
Source: Front Med (Lausanne). 2025 Jul 4;12:1588302. doi: 10.3389/fmed.2025.1588302 (PMC12271180; doi:10.3389/fmed.2025.1588302)
Supplement: Supplementary file 1 [file Table_1.docx]

**Supplementary Table S1** Parameters used in the MRI sequences at our institution (MRI Signa 1.5T Excite HD, MRI Signa 1.5T HDxt or MRI Signa Pioneer 3T GE Healthcare, Best, Netherlands)

| Sequences | TR (ms) | TE (ms) | ST (mm) | Spacing (mm) | NEX | Echo train length | Matrix |
| --- | --- | --- | --- | --- | --- | --- | --- |
| Axial T1W with and without FS* | 600-800 | 9-11 | 4-5 | 4.50-6.50 | 1 | 1 | 160-192x320-380 |
| Axial T2W FS* | 3400-4200 | 80-100 | 4-5 | 4.50-6.50 | 1 | 1 | 160-220x256-380 |
| Axial 2D MERGE*^,^** | 700 | 15 | 5-6 | 6-7 | 1 | 1 | 192x320 |
| Sagittal T1* | 600-800 | 9-11 | 4-5 | 4.50-6.50 | 1 | 1 | 160-192x320-380 |
| Sagittal T2W FS* | 3400-4200 | 80-100 | 4-5 | 4.50-6.50 | 1 | 1 | 160-220x256-380 |
| Coronal T1* | 600-800 | 9-11 | 4-5 | 4.50-6.50 | 1 | 1 | 160-192x320-380 |
| Coronal T2W FS* | 3400-4200 | 80-100 | 4-5 | 4.50-6.50 | 1 | 1 | 160-220x256-380 |
| 3 views of post-contrast T1W FS*^,^*** | 600-800 | 9-11 | 4-5 | 4.50-6.50 | 1 | 1 | 160-192x320-380 |
| Coronal T1W of the affected extremity* | 620-1160 | 9-10 | 4-5 | 4.50-5.50 | 1 | 1 | 192x320 |

*Field of view (FOV) depends on tumor size and site

**Flip angle = 20

*** The subtraction technique was routinely applied in the axial view.

TR, repetition time; TE, eco time; ST, slice thickness; NEW, number of excitations; FS, fat suppression; T1W, T1-weighted; T2W, T2-weighted; FS, fat suppression

**Supplementary Table S2** Parameters used in the MRI sequences (1.5T Magnetom, Siemens Healthcare, Erlangen, Germany)

| Sequences | TR (ms) | TE (ms) | ST (mm) | Spacing (mm) | NEX | Echo train length | Matrix |
| --- | --- | --- | --- | --- | --- | --- | --- |
| Axial T1W* | 660-700 | 9.0-9.5 | 4-5 | 4.50-6.50 | 1 | 1 | 192x320 |
| Axial STIR*^,^** | 3900-4100 | 30-45 | 4-5 | 4.00-5.50 | 1 | 1 | 192x256 |
| Sagittal T1* | 660-700 | 9.0-9.5 | 4-5 | 4.50-6.50 | 1 | 1 | 192x320 |
| Sagittal STIR*^,^** | 3900-4100 | 30-45 | 4-5 | 4.00-5.50 | 1 | 1 | 192x256 |
| Coronal T1* | 660-700 | 9.0-9.5 | 4-5 | 4.50-6.50 | 1 | 1 | 192x320 |
| Coronal STIR*^,^** | 3900-4100 | 30-45 | 4-5 | 4.00-5.50 | 1 | 1 | 192x256 |
| 3 views of post-contrast T1W FS | 700-760 | 9.00-9.60 | 4-5 | 4.00-4.50 | 1 | 2 | 192x320 |
| Coronal T1W of the affected extremity* | 660-700 | 9.0-9.5 | 4-5 | 4.50-6.50 | 1 | 1 | 192x320 |
| Coronal STIR of the affected extremity*^,^** | 3900-4100 | 30-45 | 4-5 | 4.00-5.50 | 1 | 1 | 192x256 |

*Field of view (FOV) depends on tumor size and site

**Time to inversion (TI) = 160 ms

TR, repetition time; TE, eco time; ST, slice thickness; NEW, number of excitation; FS, fat suppression; T1W, T1-weighted; STIR, Short Tau Inversion Recovery; FS, fat suppression

**Supplementary Table S3** Diagnostic accuracy at each specific cut-point in distinguishing histologic chemoresistance versus responders.

| Cut point | Sensitivity | Specificity |
| --- | --- | --- |
| 0 | 100.00% | 0.00% |
| 1 | 92.47% | 32.61% |
| 2 | 83.87% | 63.04% |
| 3 | 66.67% | 73.91% |
| 4 | 50.54% | 82.61% |
| 5 | 27.96% | 93.48% |
| 6 | 19.35% | 93.48% |
| 7 | 7.53% | 97.83% |
| 8 | 5.38% | 97.83% |

**Supplementary Table S4** Discriminative performance of the scoring model across different subgroups.

| **Subgroups** | **AuROC (95% CI)** | **AuROC difference  (95% CI)** | **P-value** |
| --- | --- | --- | --- |
| Axial location (n=16) | 0.86 (0.61, 1.00) | 0.11 (-0.12, 0.33) | 0.343 |
| Non-axial location (n=123) | 0.75 (0.65, 0.84) |  |  |
|  |  |  |  |
| Female (n=59) | 0.71 (0.54, 0.89) | -0.08 (-0.27, 0.12) | 0.437 |
| Male (n=80) | 0.79 (0.69, 0.89) |  |  |
|  |  |  |  |
| Age < 15 years (n=69) | 0.74 (0.62, 0.87) | 0.26 (-0.16, 0.21) | 0.781 |
| Age ≥ 15 years (n=70) | 0.77 (0.64, 0.90) |  |  |

**Abbreviations:** AuROC, area under the receiver operating characteristic curve; CI, confidence interval.
